# Fungal CNS Infections in Africa: The Neuroimmunology of Cryptococcal Meningitis

**DOI:** 10.3389/fimmu.2022.804674

**Published:** 2022-04-01

**Authors:** Sally H. Mohamed, Tinashe K. Nyazika, Kenneth Ssebambulidde, Michail S. Lionakis, David B. Meya, Rebecca A. Drummond

**Affiliations:** ^1^ Institute of Immunology & Immunotherapy, University of Birmingham, Birmingham, United Kingdom; ^2^ Department of Clinical Science, Liverpool School of Tropical Medicine, Liverpool, United Kingdom; ^3^ College of Health Sciences, Infectious Diseases Institute, Makerere University, Kampala, Uganda; ^4^ Fungal Pathogenesis Section, Laboratory of Clinical Immunology & Microbiology, National Institute of Allergy & Infectious Diseases, National Institutes of Health, Bethesda, MD, United States; ^5^ Institute of Microbiology & Infection, University of Birmingham, Birmingham, United Kingdom

**Keywords:** microglia, cryptococcal meningitis, fungal infection, astrocyte, HAART

## Abstract

Cryptococcal meningitis (CM) is the leading cause of central nervous system (CNS) fungal infections in humans, with the majority of cases reported from the African continent. This is partly due to the high burden of HIV infection in the region and reduced access to standard-of-care including optimal sterilising antifungal drug treatments. As such, CM is responsible for 10-15% of all HIV-related mortality, with a large proportion being preventable. Immunity to the causative agent of CM, *Cryptococcus neoformans*, is only partially understood. IFNγ producing CD4^+^ T-cells are required for the activation of myeloid cells, especially macrophages, to enable fungal killing and clearance. However, macrophages may also act as a reservoir of the fungal yeast cells, shielding them from host immune detection thus promoting latent infection or persistent chronic inflammation. In this chapter, we review the epidemiology and pathogenesis of CNS fungal infections in Africa, with a major focus on CM, and the antifungal immune pathways operating to protect against *C. neoformans* infection. We also highlight the areas of research and policy that require prioritisation to help reduce the burden of CNS fungal diseases in Africa.

## Introduction

Cryptococcal meningitis (CM) is the leading cause of fungal meningitis in humans worldwide, with the largest disease burden reported in Africa (1). The majority of CM cases are caused by members of the *Cryptococcus neoformans* and *C. gattii* species complex (2), encapsulated basidiomycetous yeasts that are prevalent in the environment, growing in soil, some plants (e.g. eucalyptus trees) and pigeon guano (3–5). CM is an AIDS defining illness, responsible for 10-15% of all HIV-related mortality globally, resulting in ~80,000 deaths annually of which nearly three-quarters (73%) occur in Africa, particularly sub-Saharan Africa where up to 60% of people with HIV reside (1). *C. neoformans* produces airborne spores that are acquired by inhalation. In healthy people, host defence mechanisms clear these spores from the alveoli in the lungs preventing symptomatic infection (6), although there is evidence to suggest that these spores may instead become dormant and reactivate during periods of immunosuppression (7). In immunocompromised hosts, these mechanisms fail allowing proliferation of *C. neoformans* in the lungs and subsequent dissemination to the CNS, causing meningitis/meningoencephalitis (6, 8). In particular, defects in T-cell immunity are highly associated with the development of CNS infection, demonstrating the important role of T-cell-mediated immunity against *C. neoformans.*

Fungal CNS infections, including CM, disproportionally affect patients in low-middle income countries, although their precise prevalence throughout the world is not well established. Global Action Fund for Fungal Infections (GAFFI) has estimated 47 million Africans suffer from fungal diseases each year (9). Across the continent, there is reduced access to gold-standard diagnostic tools and antifungal drugs for the treatment of CM (9). Moreover, it is clear that we currently have limited effective treatments for CM, since approximately one third of HIV-infected patients given antifungal prophylaxis will still go on to develop serious CNS infection (10). These worrying statistics have led to the development of a global initiative to end deaths from CM by 2030 (11), by implementing improved screening and education programs, tackling HIV management and further research into the pathogenesis of CM.

In this chapter, we discuss the epidemiology, clinical features and immunology of fungal CNS infections in Africa (focusing predominantly on CM), highlighting the areas of research that require prioritisation to help reduce the burden of these life-threatening fungal infections in Africa.

## Epidemiology of CNS Fungal Infections in Africa

Human fungal infections of the CNS are an underrepresented group of invasive infections within the African population, occurring as opportunistic infections particularly in individuals living with HIV. The most common CNS infections reported in Africa are CM and histoplasmosis (12, 13). It was estimated in 2017 that ~160,000 people were diagnosed with CM in Africa, with 98% of these cases localised to the sub-Saharan region (1). In particular, most CM cases were reported from South Africa, Nigeria and Mozambique, which averaged 20,000 cases/year/country while North Africa accounted for the least number of CM cases within the continent (1). Although recent years have seen a decrease in the yearly incidence of CM (due to improved access to antifungal and antiretroviral therapy), the mortality rates in Africa still remain high reaching 44% in short-term outcomes in routine care (14, 15) and 73% in long-term follow up studies (16–18). CM cases have been reported infrequently in children (<2% cases) with most cases found in adults living with HIV (19–22). The molecular epidemiology of *Cryptococcus* species causing CM in Africa is still not well understood, despite recent advances in technologies. *C*. *neoformans* (VNI/AFLP1) has been the major genotype causing CM in Africa, identified in >80% of isolates collected (23–30). Other *C*. *neoformans* genotypes including AFLP1B/VNII and AFLP1A/VNB have also been isolated from clinical samples and found to cause 5-10% of total CM infections (3, 25, 31–33). Increasing cases of CM as a result of *C. gattii* species complex such as *C*. *gattii* (VGI/AFLP4), *C*. *tetragattii* (VGIV/AFLP7) and *C*. *deuterogattii* (VGII/AFLP6) have been isolated in countries such as Botswana, Ivory Coast, Kenya and Zimbabwe over the past few years (25, 27, 29, 31, 33). Of note, *C*. *neoformans* (AFLP1A/VNB) and *C*. *tetragattii* (VGIV/AFLP7) are more common in Southern Africa (3, 31, 34, 35), whilst *C*. *deuterogattii* (VGII/AFLP6) has so far been only isolated in Ivory Coast (24, 29, 33).

Besides *Cryptococcus*, other human fungal pathogens are capable of invading the brain and causing CNS disease in the setting of immunodeficiency and/or traumatic or inadvertent iatrogenic inoculation into the CNS during neurosurgical procedures. The susceptibility of patients to fungal CNS infection with species other than *C. neoformans* is heavily dependent on specific risk factors, geographic location and environmental exposure. For example, CNS infection with *Candida* species is associated with CARD9 deficiency, a primary immunodeficiency caused by inherited deleterious mutations in *CARD9*. Neutrophil influx into the *Candida*-infected CNS is protective and requires CARD9 expressed by microglia (discussed below) (36, 37). CARD9 deficiency is rare, although several cases have now been reported from Africa, predominantly in Algeria (38). Interestingly, the majority of these CARD9-deficient patients shared the same mutation whereas there was greater diversity in the type of *CARD9* mutations in Asian patients (38), but whether genetic variation at the population level contributes towards the geographical distribution of invasive CNS fungal infections is unknown.

Another fungal CNS infection that has been emerging in Africa is histoplasmosis, caused by the dimorphic fungus *Histoplasma capsulatum* with the var. *duboisii* being characteristically prevalent in Africa (39, 40). This fungus is the most common pathogenic dimorphic fungus causing endemic infections in Central and West Africa and in the island of Madagascar (41). Indeed, the World Health Organisation (WHO) recently recognised histoplasmosis as a neglected tropical disease requiring further attention (9). Common risk factors for histoplasmosis include advanced HIV infection and iatrogenic immune suppression (41). CNS involvement occurs in 5-20% of patients, usually in patients with advanced infection and poor response to therapy (41, 42). Like CM, diagnosis and treatment of histoplasmosis in the African continent in hampered by availability to gold-standard diagnostic testing and antifungal drugs. Therefore, a global effort to reduce drug costs and improve accessibility will not only improve clinical outcomes in CM, but also for other life-threatening invasive fungal infections such as histoplasmosis.

## Cryptococcal Meningitis: Diagnosis, Clinical Features and Treatment

CM can be diagnosed by the identification of encapsulated yeast cells in the cerebrospinal fluid (CSF) using India Ink staining (43). However, this method can often return false negative results and is generally insensitive. Newer tests based on the detection of Cryptococcal antigen (CrAg) are much more sensitive and can allow for a rapid and low cost diagnosis (44), which is critical since many cases of CM are localised to countries with limited resources. The CrAg test works by detecting the *Cryptococcus* polysaccharide capsule antigen in the CSF; the latest versions of which are based on a lateral flow assay using an immunochromatographic dipstick. This technique is much faster and simpler than culture and/or microscopy based diagnostic assays, and can be performed at the patients’ bedside (45), and is also superior to other CrAg-based detection assays (e.g. latex agglutination assay) that require specialised laboratory equipment and skilled personnel (46). The World Health Organisation (WHO) recommends CrAg screening is performed in HIV-infected patients with a CD4 count of less than 100-200 cells/μL. A study on the effectiveness of CrAg screening in sub-Saharan Africa showed that mortality was significantly decreased when a CrAg screening program was introduced (47). Moreover, plasma CrAg titters are correlated with mortality and can lead to early identification of patients at risk of developing severe CM and death, even when symptoms are absent (10). However, several countries in Africa have limited access to the CrAg test meaning that these effective screening programs are not fully implemented in areas where they would have the greatest benefit. Therefore, improving access to these diagnostic tests is a critical step to help introduce prophylactic antifungal therapy and reduce mortality.

CM can present in the CNS as meningitis, encephalitis, or meningoencephalitis and can also result in cerebral mass lesions called “cryptococcomas” which are typically found along the perivascular spaces. CM is hard to distinguish from other types of meningitis, as there is a lack of specific clinical symptoms. In general, patients present with headache, fever, confusion and/or neck stiffness (13). Several areas of the brain can be affected by CM, including the basal ganglia, the white matter of the cerebral hemispheres, and the cerebellum (48). Computed tomography scans of CM patients usually reveal non-specific features, with ~40% of patients returning normal scans. In contrast, MRI imaging seems to perform better at assessing dilated perivascular spaces and leptomeningeal enhancement, particularly in immunocompromised patients (48), which are among the most common imaging features observed in CM patients (49).

Treatment of CM remains challenging due to the limited selection of antifungal drugs available. Even with treatment, over 70% of patients surviving CM suffer from neurological and sensory impairment, leading to disability and reduced quality of life (50). The gold standard drug for CM treatment is the combination of Amphotericin B (AMB) with flucytosine (5-FC), however a typical course of AMB and 5-FC treatment costs approximately (US)$800 per patient (50), and is usually only available in countries with well-funded healthcare systems. In Africa, only a small number of countries are registered to provide 5-FC, and even when registered there is little evidence it has been prescribed to patients in some areas. Therefore, improving the affordability of 5-FC and enhancing awareness of the drug’s effectiveness is a crucial step towards ending CM deaths (51). In addition, the use of liposomal formulations of AMB is hindered by cost. Thus, because the use of AMB-deoxycholate (AMB-d) requires prolonged hospitalization for parenteral administration and is associated with renal and metabolic adverse effects, many resource-limited settings in Africa do not use AMB for the treatment of CM. Currently, the most commonly prescribed antifungal drug for CM in Africa is fluconazole, which has been shown to be inferior to AMB (52–55). There are now several reports of fluconazole resistance developing in *C. neoformans*, associated with chromosomal changes in the fungus (56), making management of CM especially difficult in settings where alternative options are not available. Thus, novel therapeutic approaches are needed. Adjunctive immune-based therapy with interferon gamma (IFNγ) has showed promising results in recent clinical trials (57, 58). Treatment with recombinant IFNγ combined with antifungal drugs showed that the addition of recombinant IFNγ resulted in improved clearance of fungi from the CSF compared to patients treated with antifungal drugs alone, although these studies were not large enough to determine if this correlated with improved survival (57, 59). Another experimental treatment that has been suggested is the use of corticosteroids to reduce immunopathology-associated neuroinflammation (see next section), such as dexamethasone, which has been shown to reduce mortality in patients with bacterial meningitis (60). However, dexamethasone treatment for CM in HIV-infected patients actually resulted in a higher mortality rate and disability than in the placebo group, and thus these trials were suspended for safety reasons (61). We therefore still require better antifungal treatments to improve clinical outcomes in patients with CM, which will depend on a better understanding of the immunology of CM (discussed below).

## Cryptococcal Meningitis-Associated Immune Reconstitution Inflammatory Response Syndrome

HIV-associated CM management is often complicated by immune reconstitution inflammatory syndrome (IRIS) (62). In sub-Saharan Africa, where most CM infections and deaths occur, most individuals with CM have HIV infection with a profound decline in their CD4^+^ T cell counts. When antiretroviral therapy is initiated in individuals with this kind of severe immunosuppression, they undergo immune restoration albeit at varying rates (63, 64). This immune restoration occurring prior to pathogen clearance rescues pathogen specific immunity (65). These individuals then mount a pro-inflammatory response, a phenomenon termed IRIS. A similar pro-inflammatory response termed post infectious inflammatory response syndrome (PIIRS) occurs in non-HIV-associated cryptococcal meningitis (66, 67).

There are two kinds of HIV-associated CM-IRIS. First, unmasking CM-IRIS, which occurs following initiation of highly active antiretroviral therapy (HAART) in persons without any prior signs and symptoms of CM. Increased availability of HAART has not been matched by expanded CrAg screening for all individuals with advanced HIV disease, which has meant that unmasking CM-IRIS is on the increase (68). Second, paradoxical CM-IRIS, which occurs following initiation of HAART in persons previously treated for CM with documented microbiological recovery, and clinical resolution continues to decline from 20% - 30% to 3% - 6% as antifungal treatment regimens become more efficacious (69, 70). The median duration from HAART initiation to paradoxical IRIS diagnosis is 110 days (IQR, 73-227 days) (71). The main risk factors for paradoxical CM-IRIS is a high baseline CSF fungal load and a delay in CSF fungal clearance with poorly fungicidal drugs, low CD4 count, a rapid decline in HIV viral load following HAART, and early initiation of antiretroviral therapy following CM diagnosis (54, 62–69, 72, 73).

Diagnosis of CM-IRIS depends on demonstration of a rise in CSF white cell counts and protein levels, as well as evidence of inflammation on brain imaging in the setting of negative CSF fungal cultures. There is currently no definitive treatment for CM-IRIS. The recent IDSA guidelines recommend no specific treatment for minor IRIS presentation. However, for major IRIS complications manifesting with profound CSF pleocytosis and raised intracranial pressure, IDSA guidelines recommend 0.5–1.0 mg/kg per day of prednisone equivalent or higher doses of dexamethasone for severe manifestations tapered over 2 to 6 weeks (74). Although steroids have no role in treatment of active (culture positive) CM infection (see above), their use in HIV-associated IRIS is associated with improved outcomes (61, 75).

**Figure 1:**
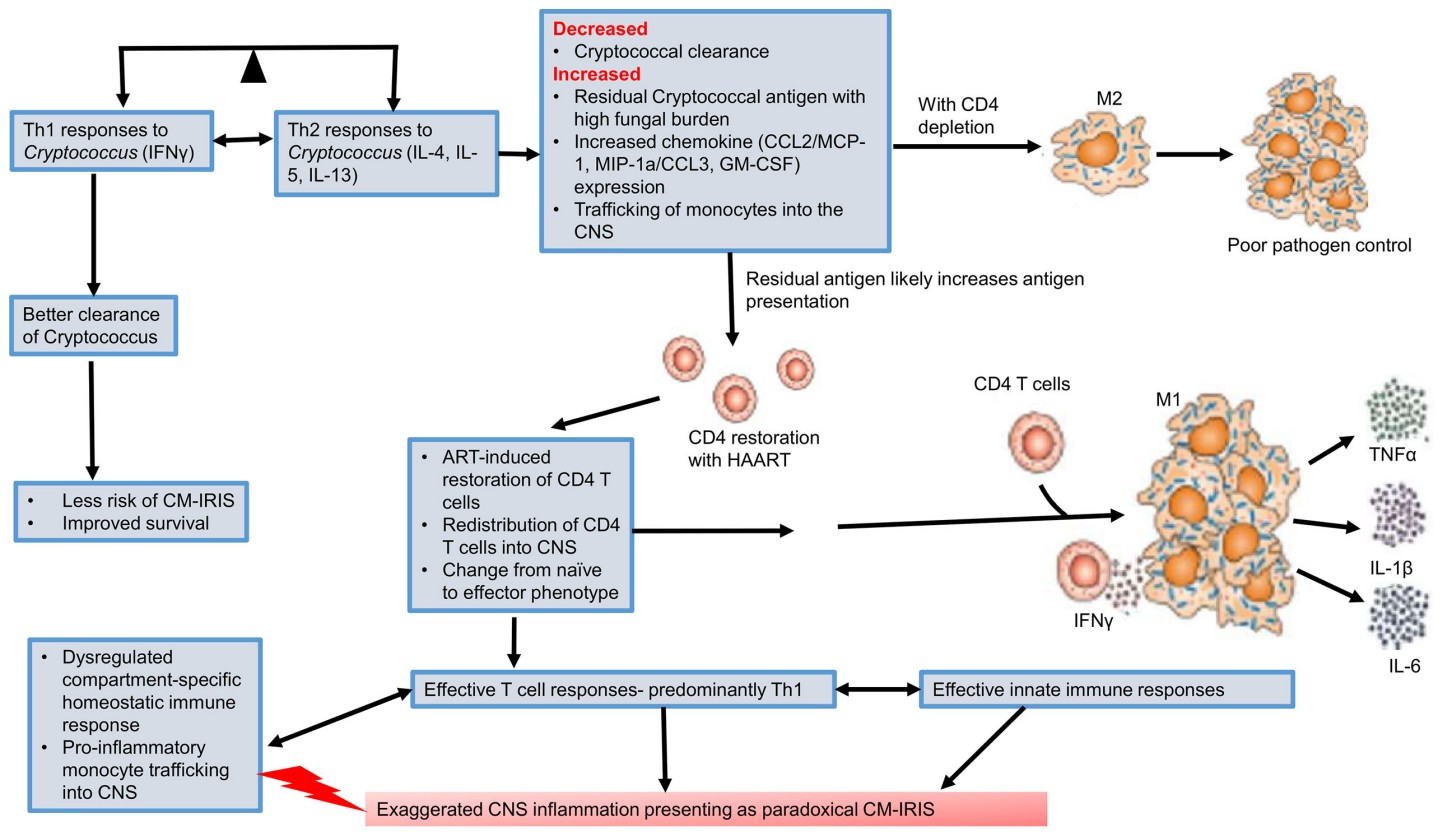
Pathogenesis of cryptococcal meningitis immune reconstitution inflammatory response syndrome (CM-IRIS). Th1 immune responses are required for better clearance of *Cryptococcus*. This reduces the risk of CM-IRIS and results in improved survival. However, there is an imbalance as the far as the Th1-Th2 paradigm is concerned with extreme HIV-associated CD4 depletion. A predominantly Th2 response is associated with M2 macrophages and poor pathogen clearance. Initiation of HAART restores CD4^+^ T cell counts. In a setting of poor pathogen clearance, the residual cryptococcal antigen induces an expansion of predominantly Th1 CD4^+^ T cells with secretion of IFNγ. This polarizes macrophages to an M1 phenotype which secretes pro-inflammatory cytokines and chemokines that recruit more innate immune cells. This predominantly Th1 immune response generates a dysregulated and exaggerated CNS inflammation that presents as paradoxical CM-IRIS. CNS, central nervous system; CM-IRIS, Cryptococcal meningitis immune reconstitution inflammatory response syndrome; HAART, highly active antiretroviral therapy.

The immunopathogenesis of paradoxical CM-IRIS is better understood than unmasking CM-IRIS as summarized here. Type 1 immune responses are driven by Th1 CD4^+^ T cells secreting IFNγ, which polarizes macrophages to an M1 phenotype associated with production of pro-inflammatory cytokines (TNFα, IL-1β, IL-12, IL-6) and enhanced synthesis of nitric oxide (**Figure 1**). As a result, M1 macrophages are highly fungicidal to phagocytosed *Cryptococcus*. In contrast, type 2 responses are driven by IL-4/13-secreting Th2 CD4^+^ T cells which polarize macrophages to an M2 phenotype, characterized by secretion of anti-inflammatory cytokines (IL-10 and TGFβ) and arginase expression, which counters nitric oxide synthesis and thus impairs clearance of *Cryptococcus* (76). The protective and non-protective roles for Th1 and Th2, respectively, may be organ-specific however, since enhanced expression of Th1 and Th2-associated cytokines are both correlated with better survival in the CSF of patients with cryptococcal meningitis (72), indicating that while Th2 is strongly associated with promoting fungal infection in the lung (72), this may not be true for the CNS.

Much as the Th1 response is appropriate for enhanced fungal clearance in both humans and murine models (see next section) (72, 77), the timing of this response and the balance with type 2 immunity is critical since dysregulated type 1 immune responses are thought to underlie the pathogenesis of IRIS (78). Current evidence shows that at paradoxical CM-IRIS diagnosis, there is a marked change in the number and phenotype of immune cells in CSF compared to when CM was diagnosed (71). For example, there is a significant increase in the number of T-cells within the CSF at the time of IRIS diagnosis, which exhibit a pro-inflammatory phenotype. Suppressive HAART rescues adaptive immune responses from the destructive effects of uncontrolled HIV replication, which had led to a decline in helper T cells. It is therefore conceivable that during paradoxical CM-IRIS, there is an increase in cryptococcal-specific peripheral blood and CSF activated (HLA-DR^+^) CD4^+^ and CD8^+^ T cells compared to when the initial CM diagnosis was made. Moreover, there is enhanced CXCR3/CXCL10 mediated signaling and trafficking of activated T cells into the CNS (79). Once within the CNS, recruited activated T cells secrete cytokines/chemokines (CCL2/MCP-1, MIP-1α/CCL3, GM-CSF) that enhance monocyte trafficking into the CNS and differentiation into inflammatory macrophages (80, 81). The recruited monocyte/macrophages are activated by *Cryptococcus*-specific Th1 cells (82). Indeed, the phenotype of CSF monocytes at the time of IRIS diagnosis has been found to have changed from a highly phagocytic classic (CD14^++^ CD16^-^) phenotype (observed at the time CM diagnosis), to more pro-inflammatory predominantly intermediate (CD14^++^ CD16^+^) and non-classical (CD14^+^ CD16^++^) phenotypes (71, 82). This shift is accompanied by an aberrant pro-inflammatory state characterized by enhanced production of TNFα, IL-1β, IL-6, and IFNγ (**Figure 1**). This exaggerated pro-inflammatory response results in damage to neurons with a rise in CSF neurofilament light chains during IRIS (83). A murine model for CM-IRIS shows that enhanced Th1 T cell infiltration in the CNS results in upregulation of astrocyte *Aqp4* mRNA, which upregulates aquaporin-4 postulated to enhance brain edema and thus neuronal injury (84).

In the context of ‘Test and Treat’, where HAART is initiated as soon as individuals have a new HIV diagnosis and in the absence of CrAg screening for those with advanced HIV disease, one area that requires more data is whether persons recently initiated on HAART (<14 days) who present with unmasking cryptococcal IRIS have a higher risk of mortality compared with persons who develop CM after more than six months of HAART (85). Understanding the mechanisms for the immunopathogenesis of unmasking IRIS should be prioritized as well as determining whether interrupting HAART in persons who develop unmasking cryptococcal IRIS could have a survival benefit.

## Cryptococcal Meningitis: Neuroimmunology

Like most invasive fungal infections, CM is largely a disease of immune-compromised patients. By studying the immune defects that promote susceptibility to CM, we are better able to understand how the mammalian immune system fights these fungal infections. This information is critical for understanding patient responses to adjunctive immune-based therapy and developing criteria to assess patient prognosis and clinical outcomes. The predominant risk factor for CM is loss of CD4 T-cells from advanced HIV infection (majority of CM cases) however there are increasing numbers of non-HIV CM being reported (66, 86). Several of these also associate with T cell dysfunction caused by various factors including lymphoma, autoimmune diseases (e.g. lupus, psoriasis, sarcoidosis), immunosuppressive therapy and idiopathic CD4^+^ lymphocytopenia (66, 87). As introduced above, T cells are essential for the activation of macrophages to kill *C. neoformans* and thus promote fungal clearance. In this section, we outline the mechanisms of fungal entry into the CNS, followed by the immunology of CM focusing on CNS-resident macrophages, astrocytes and brain-infiltrating lymphocytes, and how these different cell types contribute to protection and pathogenesis specifically within the *Cryptococcus*-infected CNS.

## *C. neoformans* Entry to the CNS

The mechanisms governing *C. neoformans* entry into the CNS are thought to be largely mediated by two main pathways, the Trojan Horse method and transcellular migration. In this section, we will outline the evidence for each of these invasion mechanisms, although it should be noted that the relative dependence on each *in vivo* for different pathologic conditions (e.g. host immunosuppression, *C. neoformans* vs *C. gattii*), is not well understood.

The Trojan horse approach involves *Cryptococcus* yeast getting access to the CNS by transporting inside phagocytic cells, such as macrophages, monocytes, and neutrophils. In support of this hypothesis, a few research studies have shown that depletion of alveolar macrophages in mice decreased the dissemination of *C. neoformans* to CNS (88, 89). Another study compared dissemination to the CNS when mice were infected with bone marrow-derived monocytes previously infected with *C. neoformans in vitro*, or with free yeast. They found that the fungal burden was higher in the brain with infected bone marrow-derived monocytes compared to free yeast cells, suggesting that infected monocytes were more efficient at disseminating infection to the CNS than free yeast (90). Indeed, depleting circulating monocytes at a later stage of infection in mice reduced infection severity and reduced fungal burden by 40% in spleen, lungs, and brain (90), thus supporting the role of phagocytes in neuroinvasion. Moreover, depleting 99% of circulating monocytes in mice before infection abolished the development of CM and cerebral cryptococcomas and reduced fungal burden in the brain by ~90% (91). Neutrophils have also been shown to potentially promote transmission to the during *Cryptococcus* infection (92). Using intravital imaging, it was shown that neutrophils can expel *C. neoformans* within the brain vasculature, contributing towards brain infection (92), and depleting circulating neutrophils resulted in a reduced number of yeast cells in the perivascular space and reduced brain fungal burden by ~ 64% (91). Finally, the Trojan horse model has been modeled *in vitro*, where cultured brain microendothelial cells were challenged with yeast-containing macrophages. This *in vitro* model showed that *C. neoformans* bound CD44 on brain endothelium *via* hyaluronic acid. Mutant strains that were unable to make hyaluronic acid (*cps1Δ*) had a profound defect in cellular transmigration (discussed below), but could be transported within macrophages indicating that Trojan Horse mediated entry can enable transport of yeast that would otherwise be restricted from the CNS (93).

Transcellular migration across brain endothelium has also been observed to promote *C. neoformans* entry into the CNS (94–96). Intra-vital microscopy experiments in mice showed that free yeast cells were able to cross capillary walls, a process that was dependent on fungal-expressed urease since blocking urease reduced transmigration into the brain (96), although it should be noted that urease also promotes intracellular survival within phagocytes (97) indicating that urease blockade might prevent fungal CNS entry by Trojan Horse as well. Other *C. neoformans* virulence factors that promote CNS entry include the metalloprotease MPR1, hyaluronic acid synthase CPS1 (as mentioned above) and transcription factor HOB1. Mutants deficient in these factors are unable to invade a model blood-brain-barrier (BBB) *in vitro*, and are avirulent in mouse infection models with a reduced capacity to establish brain infection (98) (99). In order for transcellular migration to occur, C. neoformans yeast must first be internalised by endothelial cells. Interactions between CD44 and hyaluronic acid form part of this process, but it was recently demonstrated that endothelial-expressed EphA2-tyrosine kinase receptors also play a key role (100). Inhibiting EphA2 prevented transmigration of *C. neoformans* (100), and a similar dependence on EphA2 has been observed for CNS entry by several other pathogens including *Chlamydia trachomatis*, Epstein-Barr virus, and malaria parasites (101–103), indicating that ephA2 may generally be involved with BBB permeability and pathogen entry (104).

## Microglia

**Figure 2:**
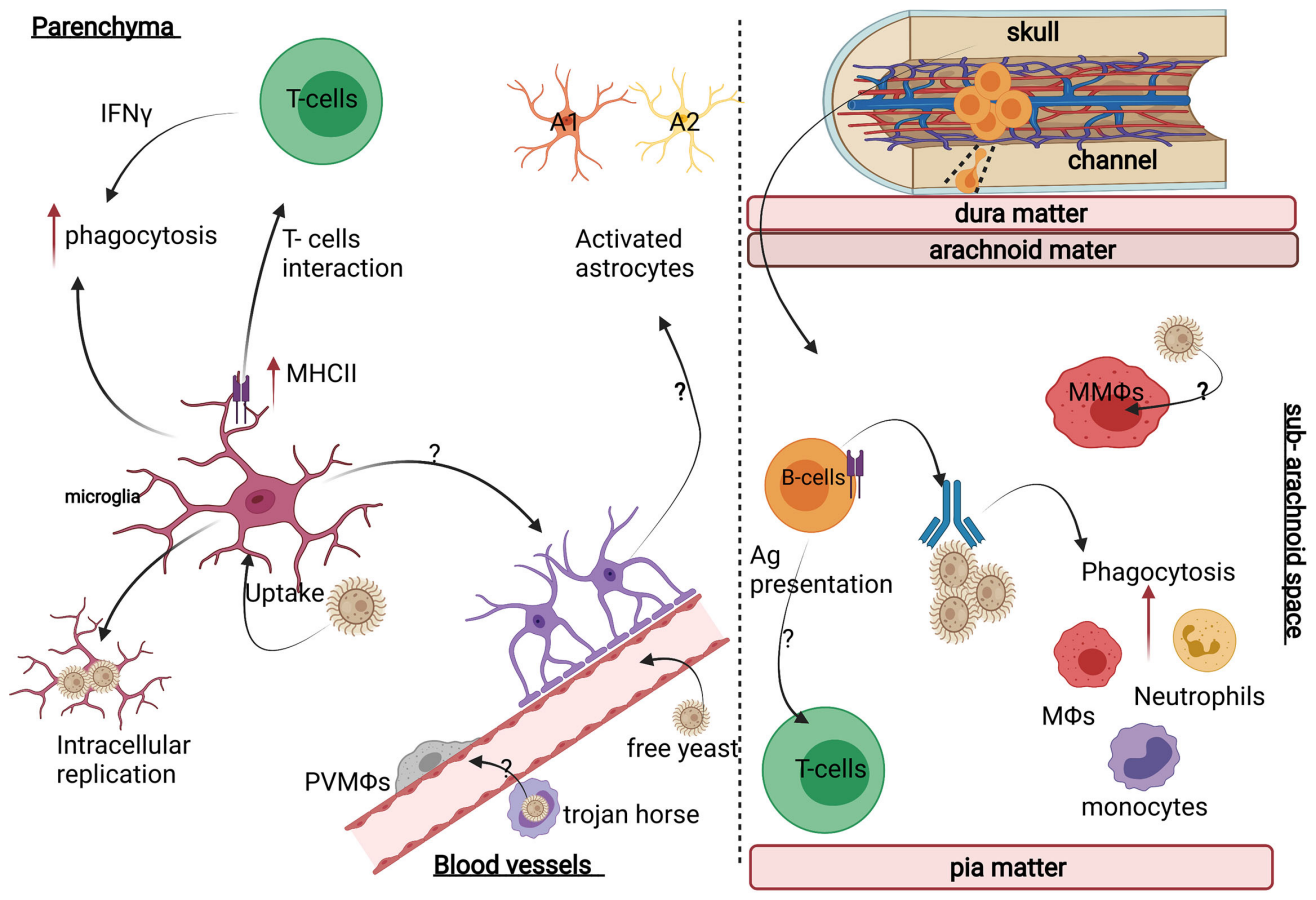
Neuroimmunology of *C. neoformans* infection. (Left panel) In the brain parenchyma, *C. neoformans* mostly interacts with brain-resident microglia and astrocytes, which differentiate into distinct functional states depending on the inflammatory context and infiltrating immune cells (e.g. T-cells). For example, astrocytes can develop into pro-inflammatory A1 or tissue-healing A2 subsets, but whether this occurs during CM is unknown. (Right panel) In the meninges, *C. neoformans* mostly localises to the sub-arachnoid space where it will encounter meningeal macrophages (MMΩs) and a variety of resident lymphocytes including B-cells (deriving from skull bone marrow and connecting channels) and T-cells. Image created with Biorender.com.

The CNS is populated by tissue-resident macrophages that exist in distinct functional subsets and localise within specific anatomical compartments. The most numerous of these CNS-resident macrophages are called microglia, which are found throughout the brain parenchyma and are involved in immune surveillance and brain development (105). Microglia are equipped with an immune arsenal to protect against brain-invading pathogens, including the expression of multiple PRRs such as the C-type lectin recptors (CLRs) and toll-like recptors (TLRs), nitric oxide synthesising enzymes and components needed to process and present antigens to CD4^+^ T cells **(** **Figure 2** **)**. *In vitro* studies showed that stimulating microglia using TLR agonists (e.g. Pam_3_ CSK_4_, LPS, and CpG) during *C. neoformans* infection drove the production of proinflammatory cytokines such as TNFα, IL-6, and IL-1β, which resulted in enhanced *C. neoformans* phagocytosis and prevented fungal intracellular replication within microglial phagosomes (106). Immortalised microglia have been shown to phagocytose *C. neoformans* leading to increased iNOS expression which is important for limiting fungal growth (107, 108). These antifungal actions are regulated by IFNγ, produced by infiltrating Th1-polarized CD4^+^ T cells. IFNγ has also been shown to induce the expression of MHC Class II by microglia *in vitro*, potentially allowing their interaction with infiltrating CD4^+^ T cells **(** **Figure 2** **)** (109–111). A study showed that immunomodulation with CD40 (a T-cell co-stimulatory molecule) and the cytokine IL-2 in *C. neoformans*-infected mice reduced the fungal burden in various organs including the brain, which correlated with an IFNγ-dependent increase of MHCII expression on microglia (112). Moreover, IFNγ knockout mice showed the critical role of IFNγ in activating microglia and inducing anti-cryptococcal activity (113, 114). Furthermore, patients with CM who feature neutralising IFNγ autoantibodies tend to have a persistent infection and lower survival rate (115). Despite these clear protective roles for microglia in controlling *C. neoformans* infections, some studies have shown that microglia are prone to latent intracellular infection, where *C. neoformans* survives and replicates inside microglial phagosomes **(** **Figure 2** **)** (116, 117). Indeed, post-mortem examinations of human brain tissue showed *C. neoformans* polysaccharide capsule is engulfed and localised inside microglia (116). Therefore, although microglia can engulf *Cryptococcus* yeast cells, the killing of yeast cells may not always occur in human microglia even when IFNγ is present (118).

Although we can gain insights into anti-cryptococcal immunity using microglia cell lines and *in vitro* models, *in vivo* studies are needed to analyse the behaviour of microglia in their natural environment since microglia rapidly lose their tissue-resident identity when removed from their microenvironment. *In vivo* studies analysing antifungal activity of microglia are so far limited. In a murine model of CM-PIIRIS, full activation of microglia did not occur until 21 days post-infection, which coincided with a significant influx of infiltrating inflammatory myeloid cells and lymphocytes and a decrease in brain fungal burdens (77). A similar observation was made following acute infection with *C. neoformans* in mice, where microglia numbers expanded >1 week post-infection which coincided with an influx of monocytes and T-cells (91). Interestingly, these effects do not occur with *C. gattii*, which demonstrates a reduced capacity for entry into the CNS compared with *C. neoformans*, with *C. gattii*-infected animals typically succumbing to significant lung disease (91). In contrast, recent *in vivo* studies showed that *C. albicans* CNS infection results in a rapid activation of microglia (within 24h), which quickly initiate protective immunity upon *C. albicans* infection. Microglia highly express CARD9 (caspase recruitment domain-containing protein 9), a signaling adaptor protein downstream of the CLRs (37). Human CARD9 deficiency results in a profound susceptibility to CNS candidiasis, aspergillosis and phaeohyphomycosis but not cryptococcal meningitis (119–121). It was recently shown that CARD9 expression by microglia is required to sense the fungal toxin Candidalysin which is secreted by *C. albicans* (36). This toxin activated the production of IL-1β and CXCL1 from microglia, which in turn recruited CXCR2-expressing neutrophils to the brain to clear the fungus (36). CARD9 deficiency does not appear to promote susceptibility to CM in humans, and deficiency in CARD9-coupled CLRs do not promote susceptibility to CM in experimental mouse models (122, 123). Thus, microglia have an important role in antifungal immunity that is context-dependent. Future studies should focus on how microglia function during CM using the latest technologies, murine models and human samples in a bid to widen our understanding of immune regulation within the *Cryptococcus*-infected CNS and how this promotes fungal clearance, as well as associated inflammatory syndromes (see IRIS section above).

## Non-Parenchymal CNS-Resident Macrophages

In addition to microglia, there are other CNS-resident macrophages found in the perivascular spaces (perivascular macrophages, PVMs), within the meninges (meningeal macrophages) and associated with the choroid plexus (choroid plexus macrophages) **(** **Figure 2** **)**. Each of these populations are poorly studied in the context of CM, with many insights into the biology of these populations only gained in recent years with the advent of new technologies (e.g. single-cell RNA sequencing) that have allowed us to better define the markers and possible functions for these cells (124–126).

Analysis of human brain autopsy tissue showed that PVMs appear to harbour intracellular *C. neoformans*, indicating that these cells interact with and phagocytose *C. neoformans* (109). Indeed, the location of PVMs would ideally position them next to the main site of infection in CM **(** **Figure 2** **)**. However, an extensive analysis of cryptococcal brain infection in mice showed that the main myeloid effector cells in the brain following *C. neoformans* infection were monocytes and neutrophils recruited from the blood, and that infection and inflammation were largely confined to the perivascular spaces where CNS-resident macrophages, including perivascular macrophages and microglia, were rare (91). Meningeal macrophages are also situated in the tissues most commonly involved in human CM. Yet, there is little research done to understand the specific contributions of these cells to fungal clearance and pathogenesis. Lastly, PVMs are the primary site for simian immunodeficiency virus (SIV) infection in the CNS, which affects the function of PVMs (127). This is important in the context of CM since it is not yet known how HIV infection (the predominant risk factor for CM in humans) affects the behaviour and function of CNS-resident macrophages such as PVMs and microglia, and the downstream consequences of this for susceptibility to cryptococcal infection. We therefore require a greater understanding of the interplay between HIV and fungal infection in these macrophage subsets and the impact of this on pathogenesis.

## Astrocytes

Astrocytes are the most numerous glial cells within the CNS and the majority of studies on astrocyte function to date have focused on their roles in maintaining neuronal health and forming a major component of the blood-brain-barrier (BBB). In recent years, new studies have revealed that astrocytes perform important immune functions and contribute towards CNS pathologies (128). During infection, astrocytes undergo a poorly understood complex process known as ‘astrogliosis’, where structural and functional changes occur. These changes are controlled by the CNS microenvironment which give rise to functionally-differentiated phenotypes that are optimised for tissue repair or resistance to infection **(** **Figure 2** **)** (129–131). Whether fungal CNS infections affect astrocyte phenotype and/or function remains an open question. One study showed that murine astrocytes undergo astrogliosis following intravenous infection with *C. neoformans* **(** **Figure 2** **)** (130), confirming that astrocytes could play roles in the pathogenesis of CM. Furthermore, *in vitro* experiments using astrocyte cell lines found that *C. neoformans* can interact with and infect human astrocytes driving an increase in MHCII expression (132, 133), providing evidence that these cells might be involved in immunity during CM. It will be worth further investigating astrocyte behaviour in CM in future studies particularly as (1) astrocytes appear to become activated during human CM and this is blunted in HIV-infected patients (134–136), and (2) astrocytes regulate traffic through the BBB thus they might have significant role in prevention of *C. neoformans* invasion of the CNS.

## T-Cells

There is growing evidence that T cells are present in the healthy CNS, which have a unique CNS-resident phenotype and are important for CNS homeostasis and animal behaviour (137–140). Mice deficient in adaptive immunity (e.g. *Rag1^-/-^*) have behavioural abnormalities, which was recently linked to their role in promoting microglial maturation in the developing brain (141–145). Moreover, T cells have been shown to promote pathology in several neurodegenerative diseases (146–148), as well as supress astrogliosis during ischaemic stroke (147). These lymphocytes are therefore integral to the outcome of CNS inflammation and important regulators of pathology in this tissue.

The specific functions of CD4^+^ T cells in the *Cryptococcus*-infected CNS remain poorly defined. As outlined above, these lymphocytes are thought to be required to activate antifungal killing pathways in myeloid cells but may also promote tissue pathology (149–151). CD4^+^ T cell recruitment to the cryptococcal-infected brain was recently shown to require CXCR3. Both human and murine T cells significantly upregulated CXCR3 in response to *C. neoformans* infection, and this chemokine receptor was required for Th1 polarization. Interestingly, *Cxcr3^-/-^* mice were protected from infection-associated CNS inflammation and thus had improved survival, but this did not correlate with reduced fungal burden. These studies therefore show that CXCR3^+^ Th1 T cells are not needed to help control fungal infection in the brain, at least in the context of an IRIS-like syndrome (152). Similarly, knockdown of CCR2 in mice was also shown to improve survival independently of fungal control in the CNS, although CCR2 was not involved in the direct recruitment of Th1 T cells to the CNS but acted indirectly by promoting the initial recruitment of inflammatory monocytes (153). Collectively, these studies indicate that T-cells have a complex role in CM, both for fungal clearance and mediating immunopathology, which is likely context- and time-dependent.

## B-Cells

B-cells produce anti-cryptococcal antibodies that are required for effective opsonisation of the fungus (particularly the capsule) and uptake by phagocytes, including macrophages (154). Patients with X-linked agammaglobulinemia (XLA), an inherited immune-deficiency caused by mutations in the *BTK* gene and characterised by an absence of B cells, have been reported to develop CM (155). Furthermore, reduced production of IgM in HIV+ patients has been correlated with a greater risk for developing CM (156). Treatment with the BTK inhibitor Ibrutinib, a drug used in the treatment of B-cell lymphomas, has been reported to promote CM in a small number of patients, although the exact underlying mechanism(s) and relative incidence of CM in Ibrutinib-treated patients remain unclear (157). Mice with B-cell and/or antibody deficiencies also have increased susceptibility to *C. neoformans* infection, characterized by higher brain fungal burden (158). Thus, B-cells provide critical support to phagocytes in the fight against CM and clearance of yeast cells from infected tissues **(** **Figure 2** **)**.

CNS border tissues, such as the meninges, were recently shown to be populated by CNS-resident B cells which infiltrated the CNS from the skull bone marrow *via* a series of bone channels **(** **Figure 2** **)**. These channels provide the meninges with a constant supply of CNS-resident B cells, which were shown to have an immunoregulatory phenotype and were optimised at recognising CNS-derived antigens (159–162). Furthermore, meningeal IgA-secreting plasma cells have been shown to curtail *Candida* invasion in the CNS (163), but whether these CNS-resident B-cells proliferate in response to *C. neoformans* infection and/or provide local protection against cryptococcal infection has not yet been determined.

## Concluding Remarks

The majority of deaths from invasive fungal infections in humans occur in Africa, and many of these are preventable. Improving access and reducing cost of ‘gold-standard’ diagnostics and treatments is urgently needed to reduce the impact of fungal CNS infections on global human health. However, even with access to antifungal drugs, mortality and morbidity from fungal CNS infection remains high. Worryingly, we are also seeing more cases of fungal CNS infections reported particularly amongst non-HIV immunosuppressed populations. It is therefore clear that we require more insights into the pathogenesis of these diseases and adjunctive immune-based therapies that boost the effectiveness of antifungal drugs. Recent advances in neuroimmunology have led to the development of models and technologies leading to novel insights into how immune responses are initiated and regulated within the CNS. Many of these models and approaches have yet to be utilised by the fungal immunology field, but their application holds significant potential in terms of discovery and future therapeutic benefit. In summary, we hope that future studies focusing on CNS antifungal immunity will shed light on how these infections may be better managed and treated, which alongside enhancing public awareness and education on the impact of fungal CNS infections, may lead to reduced mortality and improved health across Africa.

## Author Contributions

All authors wrote the manuscript, edited the final draft and approved the final submission.

## Funding

This work was supported in part by the Medical Research Council (RD), the Academy of Medical Sciences (SM, RD), the Division of Intramural Research of the National Institute of Allergy & Infectious Diseases, National Institutes of Health (ML).

## Conflict of Interest

The authors declare that the research was conducted in the absence of any commercial or financial relationships that could be construed as a potential conflict of interest.

## Publisher’s Note

All claims expressed in this article are solely those of the authors and do not necessarily represent those of their affiliated organizations, or those of the publisher, the editors and the reviewers. Any product that may be evaluated in this article, or claim that may be made by its manufacturer, is not guaranteed or endorsed by the publisher.
